# Identification of the Effects of Chondroitin Sulfate on Inhibiting CDKs in Colorectal Cancer Based on Bioinformatic Analysis and Experimental Validation

**DOI:** 10.3389/fonc.2021.705939

**Published:** 2021-09-14

**Authors:** Yingyu Zhou, Xuyang Li, Yuki Morita, Satoshi Hachimura, Takuya Miyakawa, Sachiko Takahashi, Masaru Tanokura

**Affiliations:** ^1^Department of Applied Biological Chemistry, Graduate School of Agricultural and Life Sciences, The University of Tokyo, Tokyo, Japan; ^2^Research Center for Food Safety, Graduate School of Agricultural and Life Sciences, The University of Tokyo, Tokyo, Japan; ^3^Department of Research, Medical Viara, Tokyo, Japan; ^4^Department of Research, MAF Clinic, Tokyo, Japan

**Keywords:** CDKs, colorectal cancer, bioinformatics, hub genes, chondroitin sulfate, MAP kinase pathways

## Abstract

With a high occurrence rate and high mortality, the treatment of colorectal cancer (CRC) is increasingly attracting the attention of scholars. Hub genes that determine the phenotypes of CRC become essential for targeted therapy. In the present study, the importance of cyclin-dependent kinases (CDKs) on the occurrence of CRC was identified by data mining of The Cancer Genome Atlas (TCGA) and Gene Expression Omnibus (GEO). The results showed that the gene expression levels of CDK1, CDK4, and CDK6 were obviously changed in different stages of CRC. Among the CDKs, CDK4 was suggested as an independent risk factor for CRC based on Cox analysis. Furthermore, chondroitin sulfate (CS), a kind of dietary supplement to treat osteoarthritis, was predicted to treat CRC based on its chemical structure and GEO datasets. Cell assay experiments with the human CRC cell line HCT-116 also verified this prediction. CS inhibited the gene and protein expression levels of CDKs and increased the ratios of apoptotic or dead HCT-116 cells by regulating mitogen-activated protein (MAP) kinase pathways. Our data highlight the essential roles of CDKs in CRC carcinogenesis and the effects of CS on treating CRC, both of which will contribute to the future CRC treatment.

## Introduction

Colorectal cancer (CRC) is considered the third most common and the second leading cause of death worldwide (1). The 5-year survival prognosis is highly dependent on the tumor stage of CRC: over 90% survival for stage I CRC and nearly 10% survival for stage IV CRC (2). CRC treatments consist mainly of surgery combined with adjuvant chemotherapy and neoadjuvant radiotherapy (3). However, these therapies have not been proven to effectively cure CRC with many side effects. Therefore, it is meaningful to identify hub genes and biomarkers that determine the phenotypes or tumor stages of CRC for cancer treatment.

Cyclin-dependent kinases (CDKs) are key molecules for the cell cycle. Interphase CDKs promote tumor cells to exit the G0 phase, which commits to S phase (DNA synthesis). Following S phase, DNA damage checkpoint kinases induce cell cycle arrest in G1 phase and G1-S phases (4). CDKs in mammalian cell cycle regulation, including CDK1, CDK2, CDK4, and CDK6, are usual targets for treating CRC (5). Recently, microarray technology has been widely applied to identify genetic alterations at the genome level, screen differentially expressed genes and develop novel cancer therapies (6). Along with microarray technology, bioinformatics has been processed to predict hub genes and related functional pathways (7). In the present study, two databases, The Cancer Genome Atlas (TCGA) and Gene Expression Omnibus (GEO) (8), were used to clarify the importance of CDKs in the progression of CRC. To define the functions of CDKs, gene set enrichment analysis (GSEA), Gene Ontology (GO) and Kyoto Encyclopedia of Genes and Genomes (KEGG) pathway enrichment analysis were performed. Moreover, Tetsu et al. (9) determined that inhibition of CDK2 and CDK4 leads to G1 arrest in CRC following inhibition of the mitogen-activated protein (MAP) kinase pathways, which highlights the relationships between the CDKs and MAP kinases. In addition, MAP kinase pathways, including the extracellular signal-regulated kinase (ERK), c-Jun N-terminal kinase (JNK), and p38 mitogen-activated protein kinase (p38 MAPK), were highlighted to upregulate key inflammatory mediators of cancer promoters (10, 11). These MAP kinases participate in the growth, proliferation, differentiation, and migration of various tumor cell types (10) such as pancreatic cancer, CRC, breast cancer, and gastric cancer (11).

Inhibitors of CDKs are also gaining increasing attention as novel CRC therapies (12, 13). According to the protein–ligand interaction pattern, the corresponding pharmacological effect of active molecules could be predicted (14). Based on chemical structure of active compounds and GEO datasets, we predicted that chondroitin sulfate (CS) is probably an alternative medicine to treat CRC. CS, which is extracted from terrestrial biospheres such as cows or pigs and marine biospheres such as sharks, salmon, and squid, has been reported to play a key role in the development of atherosclerosis and cancer progression *in vivo* (15). As an exogenous supplement, CS was first introduced to treat osteoarthritis (16) but has barely been reported to be a therapy for CRC. This prediction was also confirmed experimentally. We suggested that CS inhibited the gene expression levels of CDKs and increased the apoptosis or death of HCT-116 cells (a human cell line derived from colon cancer) by downregulating the MAP kinase pathways to treat CRC. Our data highlight the importance of CDKs in CRC progression and possible alternative medicines in future CRC treatment.

## Materials and Methods

### TCGA Data Mining

Clinical information was obtained from TCGA database. Gene expression (CDK1, CDK2, CDK4, and CDK6) was determined using R and Perl software (17). Gene expression levels of different tumor stages and survival rate calculations were processed by Gene Expression Profiling Interactive Analysis (GEPIA) (18).

### Cox Analysis

Cox analysis containing univariate and multivariate Cox analyses was used to select potential prognostic factors and verify the correlations between CDK expression and survival along with other clinical features (age, gender, tumor stage, T classification, M classification, and N classification) (19). *p* < 0.05 was considered statistically significant.

### GSEA

GSEA is a computational method that determines the potential function of a set of genes. To identify the potential mechanism of CDKs in CRC, “c2.cp.kegg.v7.2.symbols” was used to study the related effective pathways of CDKs (19). Gene sets with false discovery rate (FDR) *q*-values<0.05 were considered to be significantly enriched.

### GEO Data Mining

Expression profiling by arrays, including series GSE21510, GSE24514, and GSE8671, was used for GEO data mining. GSE21510 contains gene expression in colorectal cancer (CRC; n = 19) tissues and non-colorectal cancer (non-CRC; n = 25) tissues (20), GSE24514 contains 34 CRC tissues and 15 non-CRC tissues (21), and GSE8671 contains 32 CRC tissues and 32 non-CRC tissues (22). The microarray data were from the National Center for Biotechnology Information (NCBI) GEO database. The data quantity of all probes contained in the database were assessed by R software (version 3.6.3) with the R packages “affyPLM” and “affy” from the Bioconductor project and converted into the corresponding gene symbol based on annotation information in the platform GPL570 (20).

### Identification of Differential Expression Genes

The differential expression genes (DEGs) between CRC and non−CRC tissues were screened using GEO2R. |log_2_FC (fold change)| > 0.3 and *p* < 0.05 were considered statistically significant and calculated by R software with the R package “limma” from the Bioconductor project (6). The overlapping DEGs of GEO datasets were obtained by FunRich (version 3.1.3 for Windows; http://www.funrich.org/).

### Prediction of Regulated DEGs by Structure of CS

The 3D structure of CS (C_13_H_21_NO_15_S; compound CID, 24766) was obtained from PubChem. The potential target identification of CS was predicted by the PharmMapper Server using the 3D structure of CS (23) and TCMSP (the Traditional Chinese Medicine Systems Pharmacology Database and Analysis Platform) (24). A total of 1,200 genes were associated with target DEGs of CS. Two kinds of filtrating parameters, betweenness centrality (BC) and degree centrality (DC), were calculated. After filtering the top 30% BC, the top 10% DC genes were considered as the core network with Cytoscape software (National Resource for Network Biology; version 3.8.0).

### GO and KEGG Pathway Analysis

KEGG pathway analyses, GO biological process (BP), GO molecular function (MF), and GO cellular component (CC) of DEGs were predicted by the Enrichr database (25). The R packages “clusterProfiler”, “org.Hs.eg.db”, “enrichplot”, and “DOSE” from the Bioconductor project were also used to enrich the GO or KEGG pathways of DEGs. All the interactions in the present study were predicted by Cytoscape software.

### Cell Culture and Cell Viability Assay

Chondroitin sulfate was purchased from Maruha Nichiro (Tokyo, Japan). The HCT-116 cell line and Caco-2 cell line were purchased from RIKEN BioResource Research Center Cell Bank (Tsukuba, Ibaraki, Japan). The HCT-116 cell culture medium was composed of 94% Dulbecco’s modified Eagle’s medium (DMEM, Thermo Fisher Scientific, Waltham, MA, United States), 5% heat-inactivated FBS (Thermo Fisher Scientific), and 1% streptomycin (Nacalai Tesque, Kyoto, Japan). The Caco-2 cell culture medium was composed of 89% DMEM, 10% heat-inactivated FBS, and 1% streptomycin. The influences of CS on HCT-116 cell viability were determined by CCK-8 (Dojindo Molecular Technologies, Kumamoto, Japan). In brief, a density of 5 × 10^4^ HCT-116 cells/well was first seeded into 96-well flat bottom plates (Corning, New York, NY, USA) after 1 day of culture until the 60–70% area of the well was covered by cells. Different concentrations of CS (0, 0.08, 0.4, 2, 10, and 50 mg/ml) were applied to each well. After 24, 48, and 72 h incubation, the medium was removed. A total of 100 μl of CCK-8 (diluted 10 times) was put into each well. Following incubation for 30 min in an incubator (37°C, 5% CO_2_), the absorbance at 450 nm of each well was measured by a microplate reader (Tecan Trading AG, Männedorf, Switzerland). Finally, the cell viabilities under different treatments were determined by the following formula: % ratio of viable cells = [(*A*_sample_ − *A*_blank_)/(*A*_control_ − *A*_blank_)] × 100%, in which *A*_sample_ is the absorbance of each treated sample, *A*_blank_ is the absorbance of reagent only without cells, and *A*_control_ is the absorbance of the cells in the DMEM culture medium.

### Quantitative PCR

Total RNA from the cells was isolated using a QIAshredder (QIAGEN, Hilden, Germany) and RNeasy Mini Kit (QIAGEN). Quantitative PCR was performed with QuantiTect SYBR Green PCR Kits (QIAGEN) using a CFX Connect Real-Time PCR Detection System (Bio-Rad, Hercules, CA, USA). All relative gene expression levels were normalized to the gene expression level of glyceraldehyde-3-phosphate dehydrogenase (GAPDH). All the primer sequences for qPCR are shown in **Supplementary Table 1**.

### Apoptosis Assay

The apoptosis ratio was determined with an Annexin V-FITC Apoptosis Staining/Detection Kit (Abcam, Cambridge, UK). HCT-116 cells were cultured with CS (0.08 mg/ml). After 24, 48, and 72 h, the cells were washed twice with PBS(−) and resuspended in 500 μl binding buffer containing 5 μl Annexin V-FITC and 5 μl PI according to the manufacturer’s protocol. The fluorescence levels were measured by FACS Verse (BD Bioscience, Franklin Lakes, NJ, USA). All data were analyzed with FlowJo (BD Biosciences).

### Cell Protein Extracts and Immunoblotting

HCT-116 cells (6 × 10^8^) were seeded in flat-bottom 10-cm plates (Corning). After 24 h of culture (60%–70% area of plate covered by cells), 0.08 mg/ml CS was added to the plates and then cultured in an incubator (5% CO_2_; 37°C) for another 24 h. The cells were collected by 0.1% trypsin (FUJIFILM Wako Pure Chemical Corporation, Osaka, Japan), which was dissolved in EDTA-PBS(−). Protease and phosphatase inhibitor cocktail (FUJIFILM Wako Pure Chemical Corporation) was dissolved in the tissue protein extraction reagent (Thermo Fisher Scientific), which was used as protein lysis buffer. The purified protein concentration of each sample was determined by the Bradford method. Sodium dodecyl sulfate-polyacrylamide gel electrophoresis was used for the separation of all the sample proteins (20 μg). Acrylamide gel was electrophoretically transferred to polyvinylidene difluoride membrane (Millipore, Burlington, MA, USA). After blocking in 5% BSA/TBST (TBST buffer: 50 mM Tris–HCl, 150 mM NaCl, 30 mM KCl, 1% Tween-20, pH 7.5) for 1 h, the membranes were cultured overnight in the primary antibody at 4°C. The primary rabbit antibodies were β-actin, p38 MAPK, p46/54 SAPK/JNK, p44/42 MAPK (Erk1/2), phospho-p38 MAPK, phospho-p46/54 SAPK/JNK, and phospho-p44/42 MAPK (Erk1/2) (Cell Signaling Technology, Danvers, MA, USA). The primary mouse antibodies were CDK1, CDK2, CDK4, and CDK6 (Santa Cruz Biotechnology Inc., Santa Cruz, CA, USA). After that, the membranes were immersed in the bound antibody (Thermo Fisher Scientific), which stabilized goat antirabbit IgG (Thermo Fisher Scientific) for MAPKs and goat antimouse IgG (Thermo Fisher Scientific) for CDKs (1 h at room temperature). Immunoreactivity was measured by an Amersham Imager 680 (Cytiva, Marlborough, MA, USA) after dealing with sensitivity substrate (Thermo Fisher Scientific) and analyzed by the attached software (GE Healthcare Life Science, Marlborough, MA, USA).

### Cell Migration and Invasion Assay

HCT-116 cells (2 × 10^4^) were suspended in serum-free medium (300 μl) containing PBS with or without dissolved CS (0.08 mg/ml) at the upper chambers of Transwell system (Corning, 24 wells, 8-mm pore size with polycarbonate membrane). Without Matrigel (Corning) upper chambers or Matrigel-coated chambers were for migration and invasion assay, respectively. The lower chambers were filled with 750 μl 20% FBS DMEM medium. After 14 h (migration assay) or 48 h (invasion assay) incubation (5% CO_2_; 37°C), cells were fixed with methanol (FUJIFILM Wako Pure Chemical Corporation) and then stained with Crystal Violet (Cosmo Bio Co. Ltd., Tokyo, Japan). The stained cells were subsequently photographed (magnification, 40×; Olympus, Tokyo, Japan).

### Wound Healing Assay

HCT-116 cells (5 × 10^5^) were incubated in the six-well plate (Corning) overnight at 37°C, allowing cells to adhere and spread on the substrate completely. After culturing for 2 h with 10 μg/ml Mitomycin C (FUJIFILM Wako Pure Chemical Corporation), a pipet tip was used to scrape the cell monolayer in a straight line to create a “scratch”. The wounded monolayers were washed twice with PBS to remove non-adherent cells and cultured in an incubator (5% CO_2_; 37°C) containing PBS with or without dissolved CS (0.08 mg/ml). Plates were photographed at different time points, including 12, 24, 48, and 72 h (magnification, 40×; Olympus, Tokyo, Japan).

### Statistics

All the values are presented as the mean ± SEM and were analyzed by one-way ANOVA followed by Dunnett’s multiple comparison or Student’s *t*-test. A *p* < 0.05 was considered a significant difference (26).

## Results

### Gene Expression Levels of CDKs in CRC Tissues and Paracancerous Tissues Based on the TCGA Database

**Figure 1:**
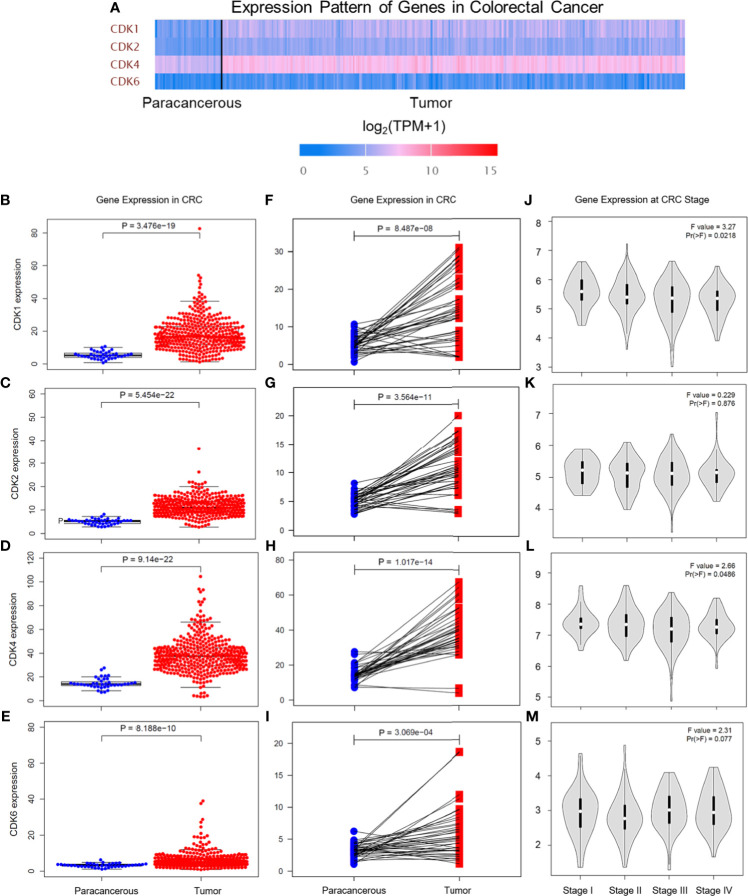
Gene expression levels of CDKs in CRC based on TCGA database. **(A)** Gene expression levels of CDK1, CDK2, CDK4, and CDK6 in paracancerous tissue and CRC tissue. TPM means transcripts per kilobase million. **(B‒E)** Gene expression levels of CDK1 **(B)**, CDK2 **(C)**, CDK4 **(D)**, and CDK6 **(E)** in paracancerous tissue (n = 39) and CRC tissue (n = 398) of CRC. **(F‒I)** Gene expression levels of CDK1 **(F)**, CDK2 **(G)**, CDK4 **(H)**, and CDK6 **(I)** in paracancerous tissue and CRC tissue from each patient. **(J‒M)** Gene expression levels of CDK1 **(J)**, CDK2 **(K)**, CDK4 **(L)**, and CDK6 **(M)** in different CRC stages.

**Figure 2:**
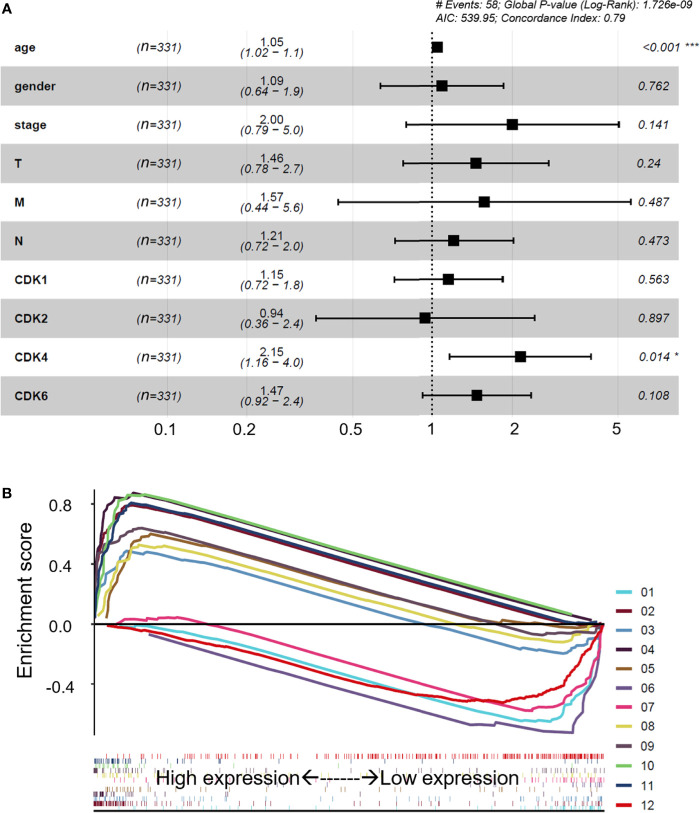
Multivariate Cox analyses and potential regulatory signaling pathways of CDKs. **(A)** Multivariate Cox analyses of clinical features and CDKs. **(B)** Related regulatory pathway prediction of CDKs based on GSEA. The pathways in the figure are as follows: 1, basal cell carcinoma; 2, cell cycle; 3, colorectal cancer; 4, DNA replication; 5, fatty acid metabolism; 6, glycosaminoglycan biosynthesis of chondroitin sulfate; 7, hedgehog signaling pathway; 8, NOD-like receptor signaling pathway; 9, p53 signaling pathway; 10, protein export; 11, RNA degradation; 12, neuroactive ligand receptor interaction. The enrichment processes of all the related pathways are shown below as bar codes. *p < 0.05 or ***p < 0.001 in the multivariate Cox analyses were considered statistically significant.

The CDK family is reported to control the tumor cell cycle and is essential for the carcinogenesis and progression of cancers (5). TCGA was used to analyze the CDKs, including CDK1, CDK2, CDK4, and CDK6. We found that in CRC tissues, the gene expression levels of CDK1, CDK2, CDK4, and CDK6 were significantly higher than the gene expression levels in paracancerous tissues (**Figure 1A**). All four CDKs had much higher expressions in the 398 CRC samples than in the 39 paracancerous samples (**Figures 1B–E**). For the paired difference analysis, CDK expression in CRC tissues was much higher than the CDK expression in paracancerous tissues from each patient (**Figures 1F–I**). Except for CD2, CDK1, CDK4, and CDK6 had obvious or inclined differential expression when comparing paracancerous tissues with stage IV CRC tissues (**Figures 1J–M**). However, the four CDKs did not show a significant influence on the survival rate of patients (**Supplementary Figure 1**), which means that although CDK1, CDK2, CDK4, and CDK6 could possibly determine the phenotypes of CRC, the CDKs could not increase the mortality rate of CRC patients. To further study the risk factors that were essential for the occurrence of CRC, univariate Cox analyses were applied to age, gender, tumor stage, T classification, M classification, N classification, and CDK family (CDK1, CDK2, CDK4, and CDK6) expressions in CRC patients (n = 331) (**Supplementary Table 2**). Among all the clinical status, the *p*-values of age, tumor stage, and tumor–node–metastasis (TMN) classification of malignant tumors were <0.05, which means that these conditions could be risk factors for overall survival among CRC patients. In addition to univariate analysis, multivariate Cox analyses were also applied to identify the independent risk factors for CRC. As shown in **Figure 2A**, the prevalence of CRC increased by 1.05 times every year, and male individuals have a 1.09 times higher prevalence than female individuals. When tumor stages and T, M, and N classifications of CRC increase 1 level, the prevalence of CRC increases by 2, 1.46, 1.57, and 1.21 times, respectively. Similarly, when the gene expression levels of CDK1, CDK2, CDK4, and CDK6 increased by one transcript per million, the prevalence of CRC increased by 1.15, 0.96, 2.15, and 1.47 times, respectively. Among all the elements, age and CDK4 expression were the two independent risk factors for the occurrence of CRC. Furthermore, to identify the potential mechanisms of CDKs in CRC, GSEA was performed to predict the CDK-related pathways with an FDR *q* < 0.05 as a filtrating value. **Figure 2B** and **Table 1** show that the cell cycle, CRC, DNA replication, fatty acid metabolism, NOD-like receptor, p53, protein export, and RNA degradation signaling pathways have positive correlations with CDKs, while basal cell carcinoma, glycosaminoglycan biosynthesis, and Hedgehog signaling pathways have negative correlations with CDKs. Especially for CDK6, in addition to the pathways downregulated by CDKs, the neuroactive ligand receptor interaction was also decreased by high CDK6 expression.

**Table 1:** The FDR *q*-values of CDKs in the KEGG pathways based on GSEA.

KEGG Name	CDK1	CDK2	CDK4	CDK6
1: Basal cell carcinoma	0.0005	0.0015	0.0010	0.0065
2: Cell cycle	0.0000	0.0000	0.0000	0.0000
3: Colorectal cancer	0.0235	0.0243	0.0322	0.0270
4: DNA replication	0.0020	0.0016	0.0018	0.0022
5: Fatty acid metabolism	0.0232	0.0205	0.0197	0.0201
6: Glycosaminoglycan biosynthesis chondroitin sulfate	0.0203	0.0171	0.0288	0.0349
7: Hedgehog signaling pathway	0.0296	0.0224	0.0326	0.0404
8: NOD like receptor signaling pathway	0.0291	0.0292	0.0327	0.0345
9: P53 signaling pathway	0.0001	0.0001	0.0002	0.0004
10: Protein export	0.0014	0.0015	0.0006	0.0009
11: RNA degradation	0.0000	0.0000	0.0000	0.0000
12: Neuroactive ligand receptor interaction	0.0610	0.0540	0.0690	0.0420

### DEG Mining Between CRC Tissues and Non-CRC Tissues Based on the GEO Database

**Figure 3:**
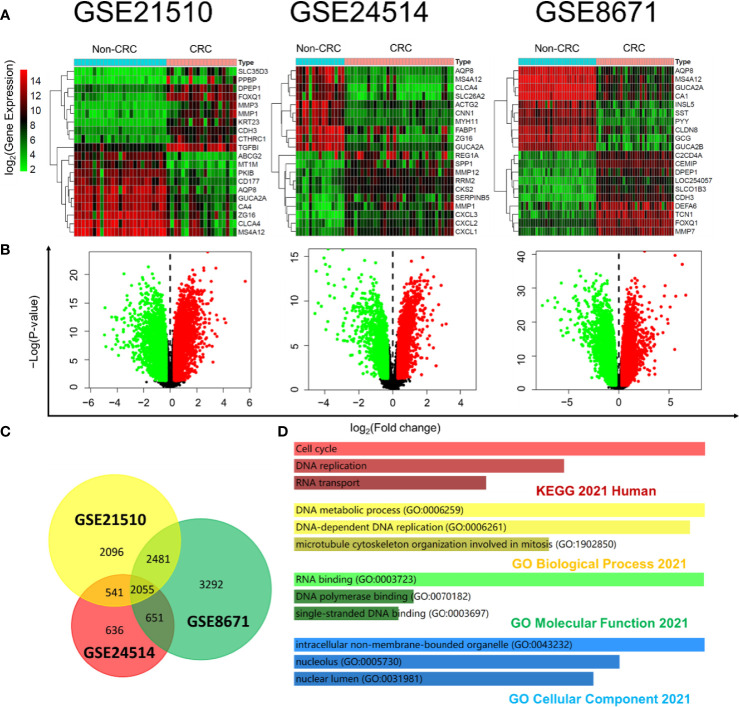
DEG mining between non-CRC tissues and CRC tissues based on the GEO database. **(A)** Heatmap of the differential gene expression in all the samples from non-CRC and CRC at the datasets GSE21510, GSE24514, and GSE8671; the color bar represents differential gene expression magnitude. **(B)** Identification of the differentially expressed genes between non-CRC and CRC at the datasets GSE21510, GSE24514, and GSE8671. (The red and green spots in the CRC were classified by the upregulated and downregulated genes compared with non-CRC, respectively.) **(C)** Venn plot of DEGs at the datasets GSE21510, GSE24514, and GSE8671 (6,544 genes were mapped in 7,173 DEGs of GSE21510; 3,790 genes were mapped in 3,883 DEGs of GSE24514; and 7,446 genes were mapped in 8,479 DEGs of GSE8671). **(D)** KEGG pathways and GO functional analysis of overlapped DEGs at the datasets GSE21510, GSE24514, and GSE8671 based on the Enrichr online tool.

Expression profiling by microarrays from series GSE21510, GSE24514, and GSE8671 was selected to help analyze the DEGs between the CRC tissues and non-CRC tissues with R software. The filtrating values to distinguish the differentially expressed genes of the three datasets were *p* < 0.05 and |log_2_FC (fold change)| > 0.3. Heatmap showed the representative gene expression levels (the top 10 highest expression and top 10 lowest expression genes in two groups) in CRC, which were significantly different from those parameters in the non-CRC group (**Figure 3A**). As shown in **Figure 3B**, the red or green spots in the CRC denote the up- or downregulated genes compared with non-CRC, respectively. A total of 2,055 overlapping DEGs (**Figure 3C**) of the three datasets were used to apply GO functional analysis and KEGG pathways with Enrichr (**Figure 3D**), and we found that the cell cycle and DNA replication were the top 2 regulated pathways, which meant that the cell cycle and DNA replication were crucial for the progression of CRC. Therefore, we searched the CDKs from the GEO microarrays. All the selected genes, including CDK1, CDK2, CDK4, and CDK6 in the three GEO datasets, showed significant differences when we compared the two groups (**Table 2**). In particular, the log_2_FC of CDK1 and CDK4 were more than 1, suggesting that these genes were possible critical genes that control the phenotypes of non-CRC or CRC. The results also clarified that CDKs expression were essential for the occurrence of CRC, which corresponded with the data from TCGA.

**Table 2:** The significance of CDKs in GEO microarrays.

Database	Gene symbol	log_2_FC	*p*-value
GSE21510	CDK1	1.84	3.75E−12
	CDK2	0.37	8.43E−11
	CDK4	2.25	1.10E−19
	CDK6	0.60	7.00E−06
GSE24514	CDK1	1.51	1.09E−10
	CDK2	0.41	2.23E−06
	CDK4	1.35	1.64E−11
	CDK6	0.20	6.87E−3
GSE8671	CDK1	1.73	1.64E−23
	CDK2	0.87	5.47E−12
	CDK4	1.10	1.24E−27
	CDK6	0.43	7.05E−07

log_2_FC, log_2_(fold change).

### The Predicted Targets of CS on CRC-Induced DEGs

**Figure 4:**
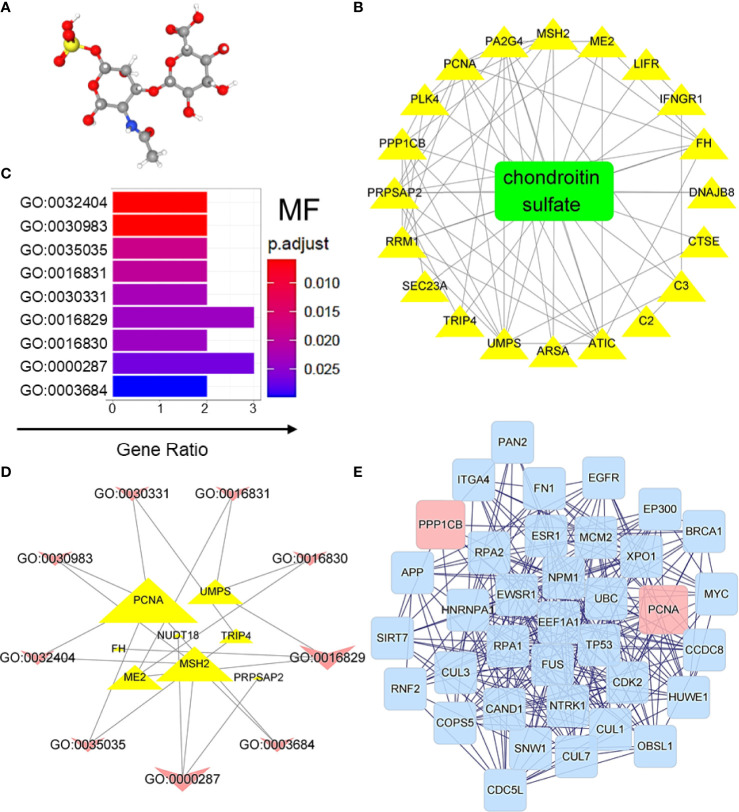
CS was predicted to treat CRC-induced DEGs. **(A)** 3D structure of CS. **(B)** Target identification of CS on CRC-induced DEGs. **(C)** Regulated GO molecular function prediction of CS on CRC-induced DEGs. GO:0032404, mismatch repair complex binding; GO:0030983, mismatched DNA binding; GO:0035035, histone acetyltransferase binding; GO:0016831, carboxy-lyase activity; GO:0030331, estrogen receptor binding; GO:0016829, lyase activity; GO:0016830, carbon-carbon lyase activity; GO:0000287, magnesium ion binding; and GO:0003684, damaged DNA binding; p.adjust, *p*-value after correction; and Gene Ratio, the proportion of genes enriched in the molecular function. **(D)** Interaction of GO molecular function and related target genes of CS. **(E)** Network topology of CS on CRC-induced DEGs. A total of 1,200 genes were associated with target DEGs of CS. Two kinds of filtrating parameters, betweenness centrality (BC) and degree centrality (DC), were calculated. After filtering the top 30% BC, the top 10% DC genes were considered as the core network.

Compared with chemical drugs, natural products have the advantages of being safe, having no side effects, and having multiple therapeutic targets; therefore, they are promising in the development of related drugs to treat various diseases. In the present study, chondroitin sulfate (CS), which was first introduced to treat osteoarthritis, was predicted to have effects on treating CRC. Based on the 3D structures (**Figure 4A**), the effective targets of CS could be predicted by PharmMapper Server and TCMSP. The overlapping DEGs of GEO samples mentioned in **Figure 3** and the predicted targets of CS could be possible targets of CS in treating CRC. The effective targets of CS and their interactions were also predicted in **Figure 4B**. Based on MF analysis (**Figure 4C**), the targets of CS on CRC-induced DEGs were highly enriched at DNA binding (GO:0030983 and GO:0003684). The interaction between the CS target genes and MF are shown in **Figure 4D**. The degree of interactions is shown by the size of delineation (genes are described by triangles, and MFs are shown by arrows). **Figure 4D** also shows that proliferating cell nuclear antigen (PCNA) had the highest affinity for MFs, and CS target genes were highly enriched at GO:0016829 (lyase activity) and GO:0000287 (magnesium ion binding). In addition, DEGs were induced by BP, and cellular component (CC) prediction of CS in CRC is shown in **Supplementary Figure 2** and **Supplementary Table 3**. Furthermore, based on the *Homo sapiens* database, the CS target genes in **Figure 4B** and their neighboring genes were analyzed. Two kinds of filtrating parameters, betweenness centrality (BC) and degree centrality (DC), were calculated. After filtering the top 30% BCs, the top 10% DC genes were considered as the core network. Finally, PCNA and protein phosphatase 1β (PP1CB) seemed to be the core genes in the network topology of predicted targets, as shown in **Figure 4E**. In particularly, PCNA is reported to be a biomarker of colonic cell proliferation (27). We also found that CS downregulated the mRNA expression levels of PCNA and PP1CB in HCT-116 cells (**Supplementary Figure 3**). The results suggest that CS might have certain effects on treating CRC.

### Effects of CS on Decreasing Expression Levels of CDKs and Related Signaling Pathways in CRC Based on Experimental Validation

**Figure 5:**
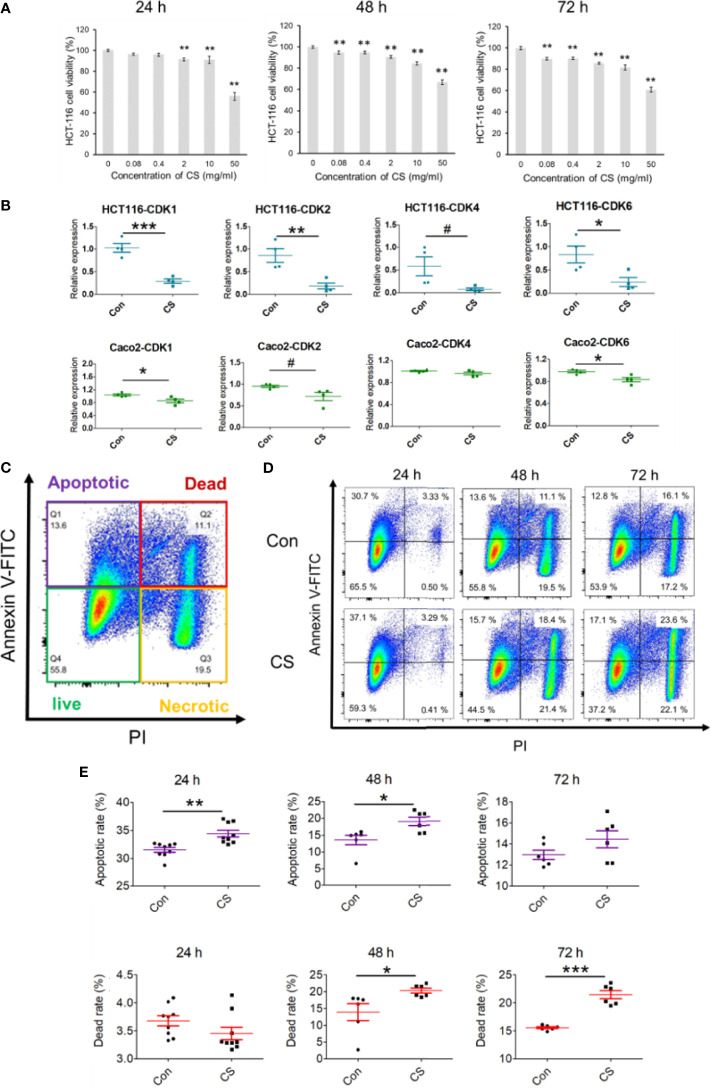
Effects of CS on decreasing the mRNA expression of CDKs and apoptosis in colorectal cancer cell lines. **(A)** The influence of different concentrations of CS (0, 0.08, 0.4, 2, 10, and 50 mg/ml) on HCT-116 cell viability after 24, 48, and 72 h (n = 4). **(B)** The effects of CS (0.08 mg/ml) on the mRNA expression levels of CDK1, CDK2, CDK4, and CDK6 after 24 h of culture (n = 4) in HCT-116 cells and Caco-2 cells. **(C)** Different cell status in the apoptosis assay with flow cytometry in HCT-116 cells. **(D)** Ratios of cell apoptosis and death after culture with CS (24, 48, and 72 h). **(E)** Histograms of cell apoptosis and death after culture with CS (24, 48, and 72 h, n = 6–9). The results are shown as the mean ± SEM. ^#^*p* < 0.1; ^*^p < 0.05; ^**^p < 0.01; ^***^p < 0.001 *versus* 0 μg/ml CS (Con) assessed using one-way ANOVA followed by Dunnett’s multiple comparison for panel **(A)**, Student’s *t*-test for panels **(B, E)** The results represent one of two independent experiments with similar results.

As all the results obtained in the bioinformatics analysis based on GEO and TCGA, CDKs were suggested to play important roles in the occurrence of CRC and CS might be a potential compound to treat CRC. A human CRC cell line, HCT-116, was used to explore the effective mechanism of CS in the present study. As shown in **Figure 5A**, CS significantly influenced HCT-116 cell viability after 24, 48, and 72 h of culture. For the 24 h coculture system of CS and HCT-116 cells, 0.08 mg/ml CS did not have a fatal influence on HCT-116 cells, which was a proper concentration to further study the antitumor mechanism of CS. The mRNA expression levels of CDK1, CDK2, CDK4, and CDK6 were significantly or tendentiously decreased by CS (0.08 mg/ml) treatment at 24 h of culture in HCT-116 cells (**Figure 5B**). We also applied another colorectal cancer cell line Caco-2 to support our results at gene levels. We found that in addition to HCT-116, CS also downregulated the expressions of CDK1, CDK2, and CDK6 in Caco-2 cells (**Figure 5B**). In addition, apoptosis assays showed that CS significantly increased the apoptotic rate of HCT-116 cells at 24 h of culture but had no influence on cell death. Meanwhile, the dead cells caused by CS were obviously increased after 48 h of culture, and the status continued after 72 h of culture (**Figures 5C–E**). The results also correspond with **Figure 5A**. The related gating processes using flow cytometry are shown in **Supplementary Figure 4**. Furthermore, we have exerted cell invasion, cell migration, and wound scratch assay to clarify the colorectal cancer cells’ proliferation ability as shown in **Supplementary Figure 5**. We found that CS had significant effects on inhibiting the proliferation abilities of HCT-116 cells.

**Figure 6:**
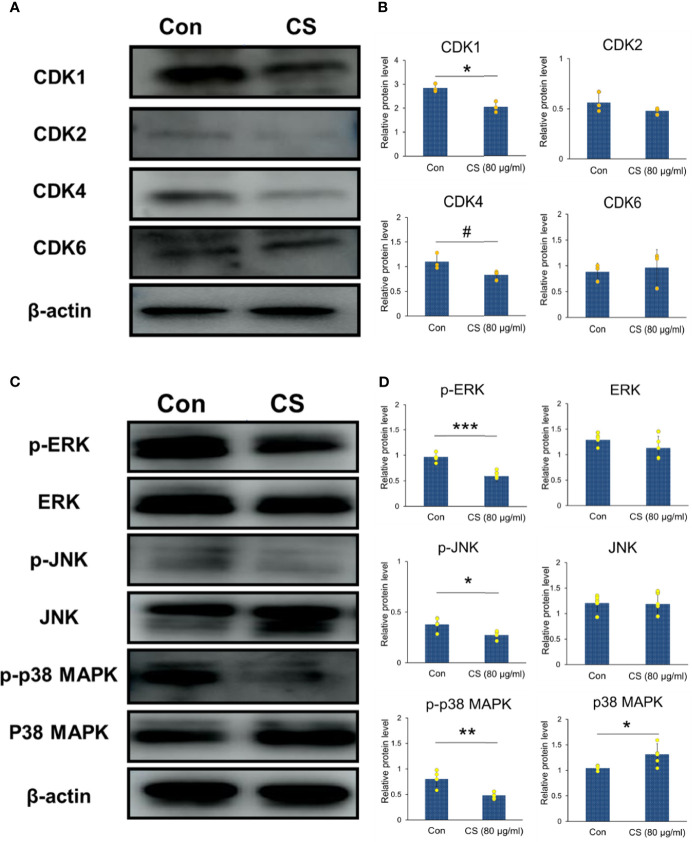
CDKs and MAP kinase regulated by CS in HCT-116 cells. **(A)** Immunoblotting of CDK1, CDK2, CDK4, CDK6 and β-actin in HCT-116 cells (β-actin was the internal control). **(B)** The protein levels of CDK1, CDK2, CDK4, and CDK6 (n = 3). **(C)** Immunoblotting of phosphorylated and unphosphorylated ERK, JNK, p38 MAPK, and β-actin in HCT-116 cells (β-actin was the internal control). **(D)** The protein levels of p-ERK, p-JNK, p-p38 MAPK, ERK, JNK, and p38 MAPK (n = 5). The results are shown as the mean ± SEM. ^#^*p* < 0.1; ^*^p < 0.05; ^**^p < 0.01; ^***^p < 0.001 *versus* 0 μg/ml CS assessed using Student’s *t*-test. The results represent one of three (CDKs) or five (MAPKs) independent experiments with similar results.

The protein levels of CDKs shown in **Figures 6A, B** were also conducted with western blotting. The results showed that CS could downregulate the protein levels of CDK1 and CDK4, which were the important DEGs based on the GEO datasets as shown in **Table 2**. In addition, CDK4 and CDK2 have been reported to inhibit G1 arrest in CRC following inhibition of the MAP kinase pathway (9). MAP kinases can influence various biological processes such as cell proliferation, inflammation, differentiation, transformation, and apoptosis. Therefore, the whole cell protein from 24 h culture of HCT-116 cells and CS (0.08 mg/ml) was subjected to Western blotting analysis to examine the effects of CS on regulating MAP kinases. The levels of the corresponding bands of phosphorylated ERK, JNK, and p38 MAPK decreased, while unphosphorylated ERK and JNK showed no significant changes (**Figures 6C, D****)**, which suggested that CS regulated MAP kinase signaling in HCT-116 cells. The results highlighted that CS was a possible candidate compound for the treatment of CRC through regulating MAP kinase signaling pathways and decreasing the mRNA expression and protein levels of CDKs.

## Discussion

With low consumption and high accuracy, a growing attention has been focused on bioinformatics to apply hub genes prediction, which would be biomarkers for disease treatment. In the present study, TCGA and GEO were performed to help us to understand the underlying mechanisms of CDKs in the production of CRC. CDKs are essential proteins in regulating epigenetic modifications, interrupting DNA-damage responses, and participating in cell cycle machinery (28). Abnormal activation of CDKs would promote the development of malignant cancer (29). However, the significant roles of CDKs during the CRC procession from big data perspective are not yet elucidated. In the study, multivariate Cox analyses were applied to identify the risk factors for CRC. We found that among all the elements (including age, gender, tumor stages T, M, and N classifications of CRC, and gene expressions of CDKs), age and CDK4 expression were the two independent risk factors for the occurrence of CRC. That means CDK4 would be an independent biomarker, which would be helpful for the clinical diagnosis of CRC. Although CDKs did not influence the survival rate of CRC patients, it does not mean CDKs were not meaningful. The CDKs, including CDK1, CDK2, CDK4, and CDK6 in the three GEO datasets (GSE21520, GSE24514, and GSE8671), showed significant difference when we compared the non-CRC and CRC groups (**Table 2**). In particular, the log_2_FC values of CDK1 and CDK4 were more than 1, suggesting that these genes were probably critical genes that control the phenotypes of the two groups. The results also clarified that CDKs expression was essential for the occurrence of CRC, which agreed with the data from TCGA (**Figures 1**, **2**). In addition, GSEA was applied to determine the underlying pathways regulated by CDKs in CRC. We found that besides the cell cycle, DNA replication, fatty acid metabolism, protein export, and RNA degradation signaling pathways, the NOD-like receptor and p53 pathways were also regulated. NOD-like receptors such as NLRP2 (30), NLRP6 (31), and NLRP12 (32) are widely reported to play important roles in colon inflammation and tumorigenesis. Meanwhile, p53 is a substrate of serine kinases such as JNK, p38 MAPK, and ERK (33). As an anticancer target, p53 elicits cellular responses to various cellular stresses and further affects DNA repair, cell cycle arrest, senescence, and apoptosis (34). All the results mentioned above suggested the essential roles of CDKs in the carcinogenesis of CRC.

Drugs targeting CDKs have been conducted for the treatment of various tumors (35). CDK2 and CDK4 have been reported to inhibit G1 arrest in CRC following inhibition of the MAP kinase pathways, which suggests interrelation between CDKs and MAP kinases (9). In addition, among the MAP kinases, the activation of the JNK and p38 MAPK pathways influences the tumorigenesis-related functions of tumor cells, alters the tumor microenvironment, and plays essential roles on crosstalk with other signaling pathways (11). There is also growing evidence that activation of MAP kinases promotes pathogenesis, progression, oncogenic behavior, invasion, and metastasis of CRC (36). Based on the chemical structures of small molecular compounds, CS, a sulfated glycosaminoglycan used in dietary supplements as an alternative medicine to treat osteoarthritis, was predicted to be capable of treating CRC. Potential drug target identification of CS suggested that CS may regulate PCNA and PPP1CB. PCNA seems to be a new marker to study human colonic cell proliferation (27), and high PCNA expression has prognostic significance in colon adenocarcinoma (37). These predictions also supported that CS impacts CRC formation and progression. Furthermore, to clarify the effects of CS on CRC, experimental validations that related to HCT-116 cell line were applied. We found that CS decreased the mRNA and protein expression levels of CDKs and inhibited the proliferation abilities of HCT-116 cells. Meanwhile, CS significantly increased the ratios of apoptotic or dead HCT-116 cells by regulating the ERK, JNK, and p38 pathways. The results suggested that CS might have effects on treating CRC.

## Conclusions

The present study presented the importance of CDKs in colorectal cancer procession based on the GEO and TCGA databases. Among all the CDKs, including CDK1, CDK2, CDK4, and CDK6, CDK4 was defined as an independent risk factor for colorectal cancer. In addition, chondroitin sulfate was suggested to be a possible medicine that can treat CRC by regulating MAP kinase signaling pathways and decreasing the mRNA expression and protein levels of CDKs. The study provides that CS is a potential candidate for CRC treatment and the basic evidence for a future *in vivo* study.

## Data Availability Statement

The raw data supporting the conclusions of this article will be made available by the authors, without undue reservation.

## Ethics Statement

All the experimental protocols were approved by the Experimental Ethics Committee of the Graduate School of Agricultural and Life Sciences of the University of Tokyo.

## Author Contributions

MT and ST conceived this study. YZ, XL, and MT designed the research studies. YZ, XL, and YM conducted the experiments. YZ analyzed the data assisted by SH, TM, and MT. YZ, SH, TM, and MT wrote the manuscript. All authors contributed to the article and approved the submitted version.

## Funding

This research was supported by the collaboration fund (number 180600000690) from Medical Viara.

## Conflict of Interest

The authors declare that this study received funding from Medical Viara. The funder had the following involvement in the study: YM partially assisted to conduct the experiments of YZ and XL.

The remaining authors declare that the research was conducted in the absence of any commercial or financial relationships that could be construed as a potential conflict of interest.

## Publisher’s Note

All claims expressed in this article are solely those of the authors and do not necessarily represent those of their affiliated organizations, or those of the publisher, the editors and the reviewers. Any product that may be evaluated in this article, or claim that may be made by its manufacturer, is not guaranteed or endorsed by the publisher.
