# Supplementary material for: Identification of the Effects of Chondroitin Sulfate on Inhibiting CDKs in Colorectal Cancer Based on Bioinformatic Analysis and Experimental Validation
Source: Front Oncol. 2021 Sep 14;11:705939. doi: 10.3389/fonc.2021.705939 (PMC8477652; doi:10.3389/fonc.2021.705939)
Supplement: Supplementary file 1 [file Image_1.pdf]

Supplementary Material

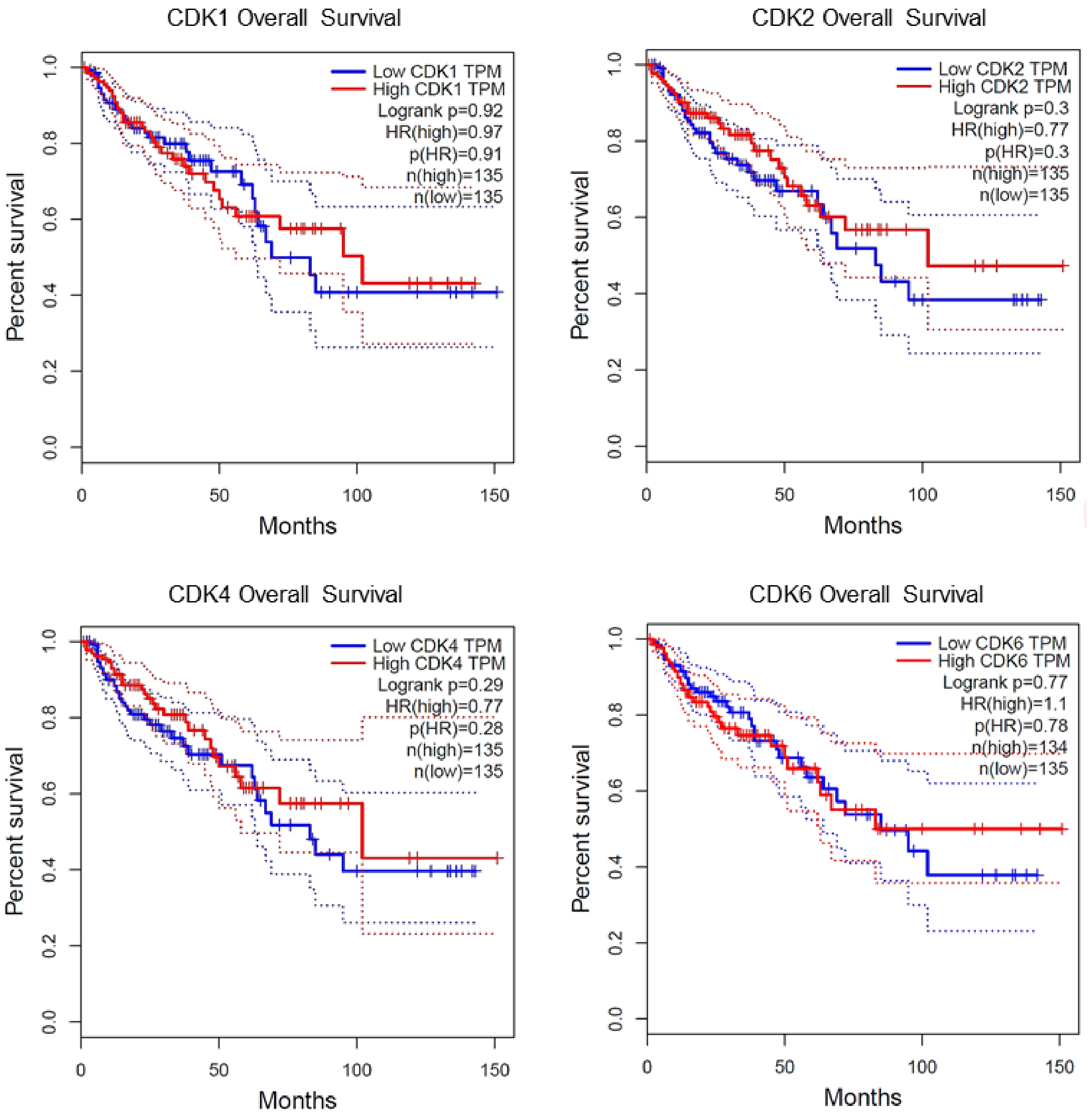

Supplementary Figure 1. Influences of the gene expression levels of CDK1, CDK2, CDK4 and CDK6 on the survival rate of patients (full lines are the overall survival, and dotted lines are the 95% confidence intervals).

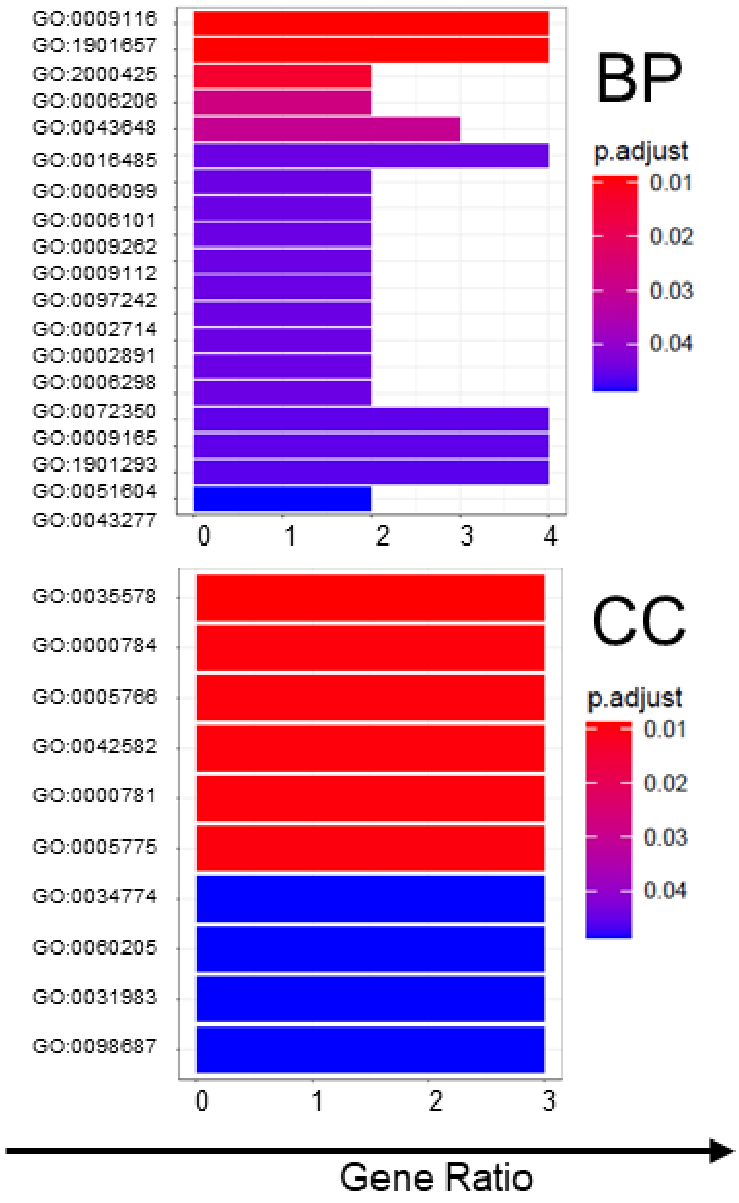

Supplementary Figure 2. GO biological process (BP) and cellular component (CC) prediction of CS on CRC-induced DEGs. All the GO descriptions are shown in **Supplementary Table 3** (*p*.adjust, *p*-value after correction; and Gene Ratio, the proportion of genes enriched in the biological processes).

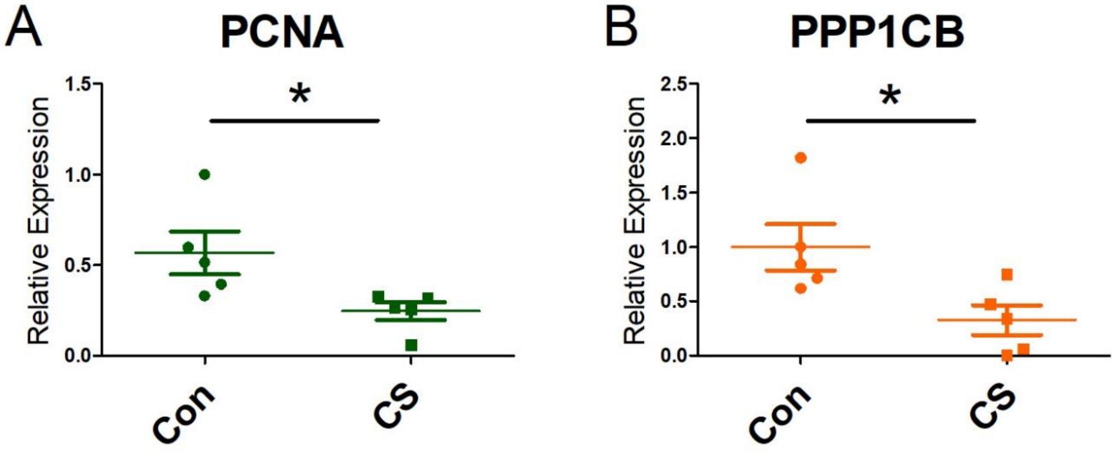

Supplementary Figure 3. Effects of CS on regulating the mRNA expression levels of PCNA and PPP1CB in HCT-16 cells (n=5). (**A**) PCNA mRNA expression level of HCT-16 cells. (**B**) PPP1CB mRNA expression level of HCT-16 cells. $^{*}p<0.05$ versus 0 μg/ml CS assessed using Student's *t*-test. The results represent one of two independent experiments with similar results.

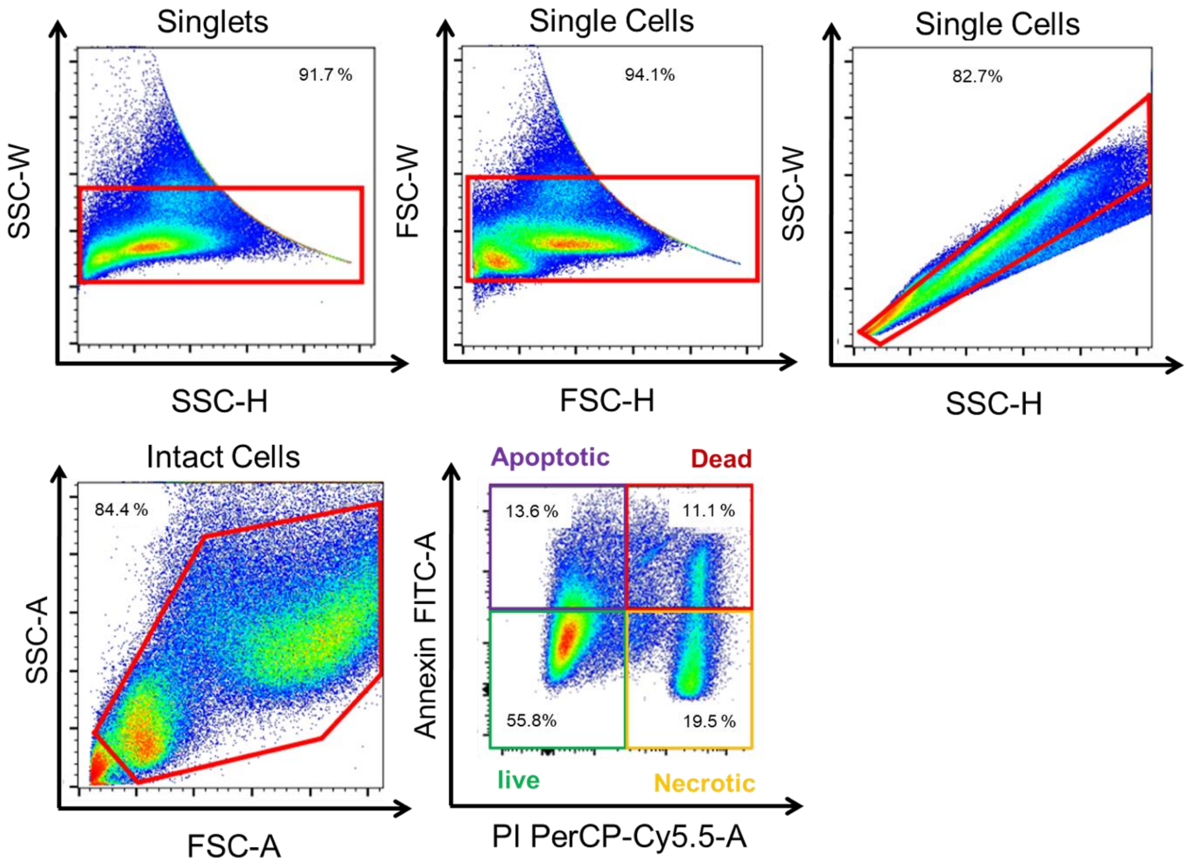

Supplementary Figure 4. Analysis of apoptosis using flow cytometry. FSC, forward scatter; and SSC, side scatter.

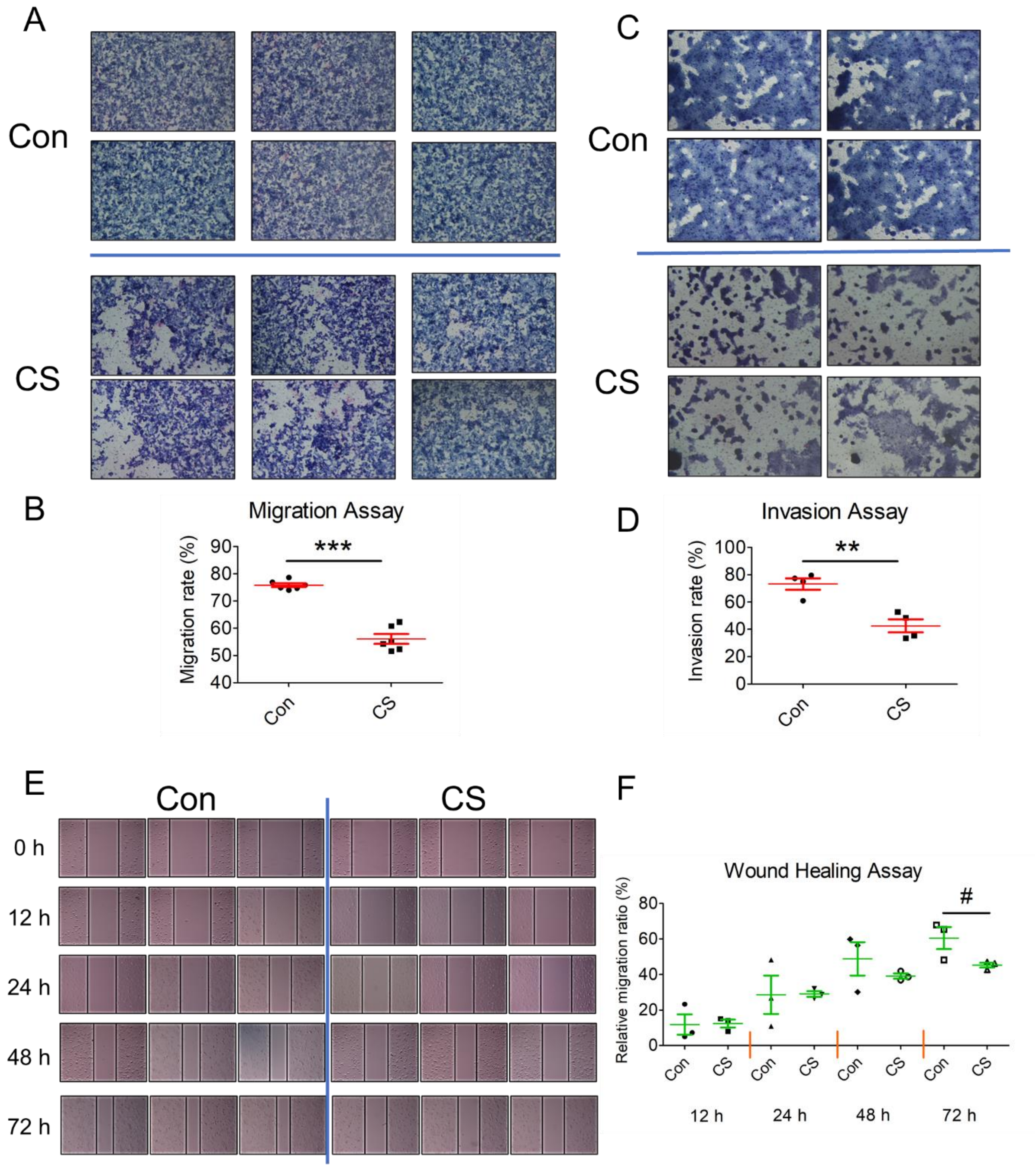

Supplementary Figure 5. Effects of CS on inhibiting the proliferation abilities of HCT-116 cells. (**A**) Photographs of migrated HCT-116 cells (magnification × 40). (**B**) CS inhibited the migration abilities of HCT-116 cells (n=6). (**C**) Photographs of invaded HCT-116 cells (magnification × 40). (**D**) Inhibition of the invasion abilities of HCT-116 cells (n=6). (**E**) Photographs of migrated HCT-116 cells in wound heling assay (magnification × 40). (**F**)Inhibition of the migration abilities of HCT-116 cells in wound heling assay (n=3). The results are shown as the mean±SEM. $^{\#}p<0.1$; $^{**}p<0.01$; $^{***}p<0.001$ versus 0 µg/ml CS assessed using Student's *t*-test. The results represented one of two independent experiments with similar results.
