# Supplementary material for: Identification of the Effects of Chondroitin Sulfate on Inhibiting CDKs in Colorectal Cancer Based on Bioinformatic Analysis and Experimental Validation
Source: Front Oncol. 2021 Sep 14;11:705939. doi: 10.3389/fonc.2021.705939 (PMC8477652; doi:10.3389/fonc.2021.705939)
Supplement: Supplementary file 2 [file Table_1.pdf]

Supplementary Material

Supplementary Table 1. Primer sequences for qPCR.

Targets	qPCR primers
CDK1	5'- AAACTACAGGTCAAGTGGTAGCC-3' (forward)
	5'- TCCTGCATAAGCACATCCTGA-3' (reverse)
CDK2	5'- CCAGGAGTTACTTCTATGCCTGA-3' (forward)
	5'- TTCATCCAGGGGAGGTACAAC-3' (reverse)
CDK4	5'- ATGGCTACCTCTCGATATGAGC-3' (forward)
	5'- CATTGGGGACTCTCACACTCT-3' (reverse)
CDK6	5'- GCTGACCAGCAGTACGAATG-3' (forward)
	5'- GCACACATCAAACAACCTGACC-3' (reverse)
PPP1CB	5'-TGTCCCTGATACAGGTTTGCT-3' (forward)
	5'-AACTCGCCACAGTAATTTGGG-3' (reverse)
PCNA	5'-CCTGCTGGGATATTAGCTCCA-3' (forward)
	5'-CAGCGGTAGGTGTCGAAGC-3' (reverse)

Supplementary Table 2. Univariate analysis of the correlation of the CDK family with overall survival among colorectal cancer patients.

Elements	HR	HR.95L	HR.95H	*p*-value
age	1.02987	1.00509	1.05526	1.79E-02
gender	0.88771	0.52653	1.49669	6.55E-01
tumor stage	2.50188	1.85077	3.38205	2.48E-09
T	2.92672	1.74213	4.91678	4.97E-05
M	5.22594	3.06475	8.91115	1.25E-09
N	2.17526	1.60888	2.94101	4.41E-07
CDK1	1.13618	0.80529	1.60304	4.67E-01
CDK2	1.26568	0.68008	2.35553	4.57E-01
CDK4	1.12616	0.68241	1.85849	6.42E-01
CDK6	1.40469	0.92881	2.12440	1.07E-01

HR, hazard ratio; HR.95L, lower limit of 95% confidence interval; and HR.95H, upper limit of 95% confidence interval

Supplementary Table 3. GO description.

GO ID	Description
GO:0009116	nucleoside metabolic process
GO:1901657	glycosyl compound metabolic process
GO:2000425	regulation of apoptotic cell clearance
GO:0006206	pyrimidine nucleobase metabolic process
GO:0043648	dicarboxylic acid metabolic process
GO:0016485	protein processing
GO:0006099	tricarboxylic acid cycle
GO:0006101	citrate metabolic process
GO:0009262	deoxyribonucleotide metabolic process
GO:0009112	nucleobase metabolic process
GO:0097242	amyloid-beta clearance
GO:0002714	positive regulation of B cell mediated immunity
GO:0002891	positive regulation of immunoglobulin mediated immune response
GO:0006298	mismatch repair
GO:0072350	tricarboxylic acid metabolic process
GO:0009165	nucleotide biosynthetic process
GO:1901293	nucleoside phosphate biosynthetic process
GO:0051604	protein maturation
GO:0043277	apoptotic cell clearance
GO:0035578	azurophil granule lumen
GO:0000784	nuclear chromosome, telomeric region
GO:0005766	primary lysosome
GO:0042582	azurophil granule
GO:0000781	chromosome, telomeric region
GO:0005775	vacuolar lumen
GO:0034774	secretory granule lumen
GO:0060205	cytoplasmic vesicle lumen
GO:0031983	vesicle lumen
GO:0098687	chromosomal region
